# Integration of nano‐ and biotechnology for beta‐cell and islet transplantation in type‐1 diabetes treatment

**DOI:** 10.1111/cpr.12785

**Published:** 2020-04-27

**Authors:** Andras Dinnyes, Andrea Schnur, Suchitra Muenthaisong, Peter Bartenstein, Charles‐Thibault Burcez, Neal Burton, Clemens Cyran, Pierre Gianello, Elisabeth Kemter, Gabor Nemeth, Francesco Nicotra, Eszter Prepost, Yi Qiu, Laura Russo, Andras Wirth, Eckhard Wolf, Sibylle Ziegler, Julianna Kobolak

**Affiliations:** ^1^ Biotalentum Ltd Hungary Godollo Hungary; ^2^ Sichuan University College of Life Sciences Chengdu China; ^3^ Department of Dermatology and Allergology Research Institute of Translational Biomedicine University of Szeged Szeged Hungary; ^4^ Department of Nuclear Medicine Faculty of Medicine Ludwig‐Maximilians University Munchen Germany; ^5^ Defymed Strasbourg France; ^6^ iThera Medical GmbH Munchen Germany; ^7^ Department of Clinical Radiology Faculty of Medicine Ludwig‐Maximilians University Munchen Germany; ^8^ Health Science Sector ‐ Laboratory of Experimental Surgery and Transplantation Université Catholique de Louvain Brussels Belgium; ^9^ Faculty of Veterinary Medicine Gene Center and Department of Biochemistry Ludwig‐Maximilians University Munchen Germany; ^10^ Mediso Medical Imaging Systems Budapest Hungary; ^11^ Department of Biotechnology and Biosciences University of Milano‐Bicocca Milan Italy; ^12^ BBS Nanotechnology Ltd Debrecen Hungary

## Abstract

Regenerative medicine using human or porcine β‐cells or islets has an excellent potential to become a clinically relevant method for the treatment of type‐1 diabetes. High‐resolution imaging of the function and faith of transplanted porcine pancreatic islets and human stem cell–derived beta cells in large animals and patients for testing advanced therapy medicinal products (ATMPs) is a currently unmet need for pre‐clinical/clinical trials. The iNanoBIT EU H2020 project is developing novel highly sensitive nanotechnology‐based imaging approaches allowing for monitoring of survival, engraftment, proliferation, function and whole‐body distribution of the cellular transplants in a porcine diabetes model with excellent translational potential to humans. We develop and validate the application of single‐photon emission computed tomography (SPECT) and optoacoustic imaging technologies in a transgenic insulin‐deficient pig model to observe transplanted porcine xeno‐islets and in vitro differentiated human beta cells. We are progressing in generating new transgenic reporter pigs and human‐induced pluripotent cell (iPSC) lines for optoacoustic imaging and testing them in transplantable bioartificial islet devices. Novel multifunctional nanoparticles have been generated and are being tested for nuclear imaging of islets and beta cells using a new, high‐resolution SPECT imaging device. Overall, the combined multidisciplinary expertise of the project partners allows progress towards creating much needed technological toolboxes for the xenotransplantation and ATMP field, and thus reinforces the European healthcare supply chain for regenerative medicinal products.

## 1 INTRODUCTION

Diabetes is one of the most challenging and economically important areas in medicine. Current data from the International Diabetes Federation indicate that 425 million people worldwide suffer from diabetes, and this is predicted to increase to 629 million people by 2045 (https://www.idf.org/diabetesatlas). About 10% of these subjects suffer from type‐1 diabetes (T1D), which involves immune‐mediated destruction of the insulin‐producing pancreatic beta cells and thus requires exogenous insulin replacement.

Despite remarkable advances in recent years in treatment of diabetes, with technical developments,^1^ T1D patients develop frequent hypoglycaemic episodes and extreme glycolic lability. For patients with recurrent life‐threatening hypoglycaemia, pancreas or islet transplantation is considered as ultimate therapeutic option,^2^ which are able to normalize glucose homeostasis and prevent macro‐ and microvascular complications of diabetes. However, requirement of lifelong immunosuppression and the critical lack of human donor organs are major limitations that urgently call for alternative options including stem cell–derived insulin‐producing cells and xenogeneic islets.^3^, ^4^, ^5^ Xenotransplantation of cells, tissues and organs is a rapidly developing field.^6^, ^7^, ^8^ The pig is the favourite donor species for several reasons, including similarity with humans in the size and physiology of many organs, high fecundity and the possibility of genetic modification. However, its major limitation is the need for immunosuppressive treatment to prevent graft rejection which have significant side effect such as diminishing islet function or the induction of islet death. Immune isolation of cells by encapsulation with a permselective membrane, which protects cells against the recipient's immune system, but allows nutrient and oxygen supply,^9^, ^10^ is a promising strategy for allo‐ and xenogenic cell transplantation. This avoids lifelong immunosuppression and should be readily implemented in the clinic.

An alternative and promising therapeutic strategy is based on the generation of new human beta cells by directed differentiation of pluripotent stem cells (either embryonic stem cells (ESCs) or induced pluripotent stem cells (iPSCs)) through the various stages of beta‐cell development into endocrine progenitors or functional beta cells.^11^ Different strategies to generate new beta cells are currently being discussed.^12^ Clinical trials with products from pluripotent stem cells have begun (eg Clinicaltrials.gov: NCT02239354, NCT03163511) but clinical efficacy still needs to be proven. Detection and monitoring the fate of cell and tissue transplants in vivo are of utmost importance for development of clinical cell therapy.

**Figure 1:**
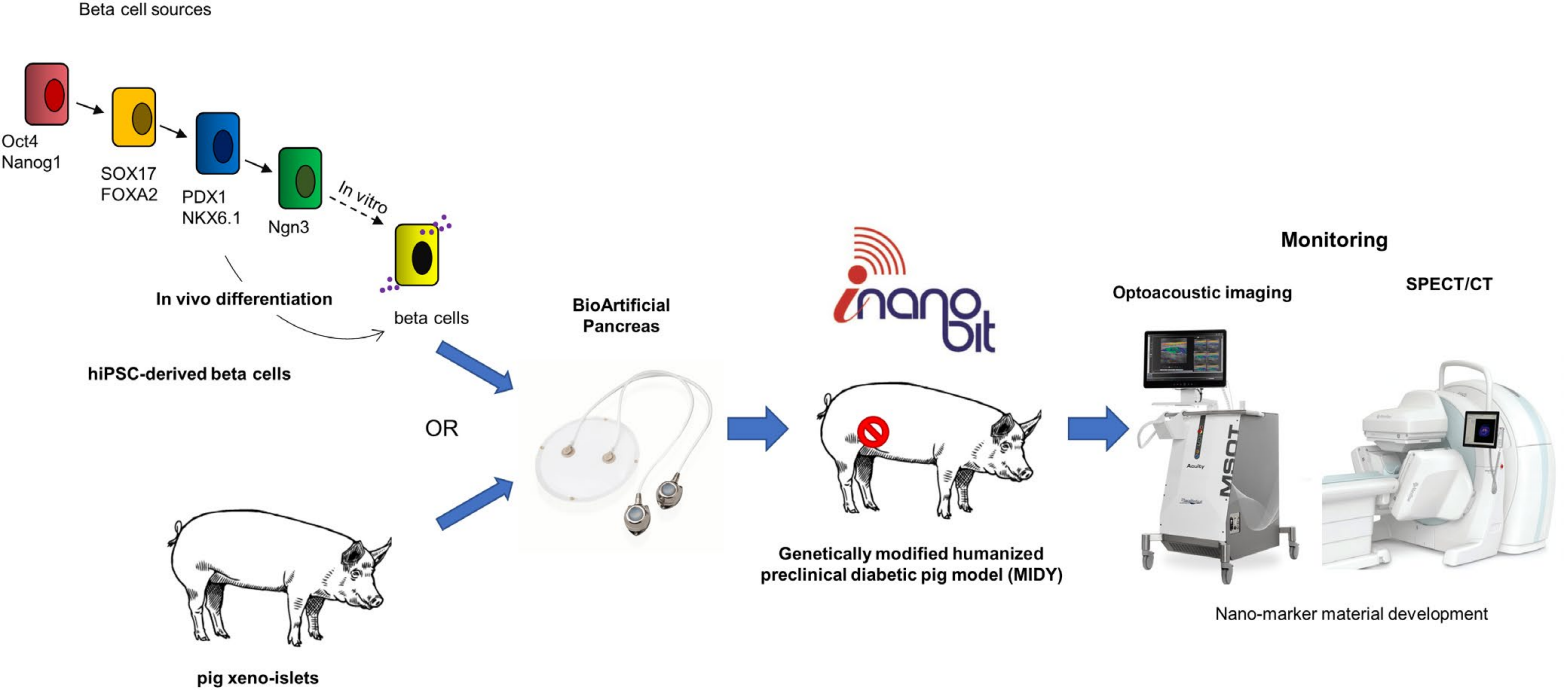
The structure and toolboxes of the iNanoBIT project. iRFP expressing xeno‐islets and hiPSC‐derived beta cells will be transplanted in genetically modified humanized pre‐clinical diabetic pig model. Highly sensitive nanotechnology‐based imaging approaches (Optoacoustic imaging, SPECT/CT) will be designed to monitor of survival, engraftment, proliferation, function and whole‐body distribution of the cellular transplants

Recent years of accelerated technological development in imaging technologies both in small animals and clinical imaging allows an unprecedented growth in applications. Yet, the resolution and quantification accuracy obtained in small animal models for PET, SPECT, CT, MR and optoacoustic imaging are not yet replicable in large animal models and in clinical settings. The aims of the iNanoBIT project^13^ are to develop and validate the application of state‐of‐the art imaging technologies and to invent novel and highly sensitive nanotechnology‐based imaging approaches allowing monitoring of survival, engraftment, proliferation and whole‐body distribution of functional cellular transplants (xeno‐islets or human beta cells derived from human iPSCs) as well as the subsequent regenerative processes in vivo in a genetically diabetic pre‐clinical porcine model. (Figure 1) The present work‐in‐progress review will provide a short overview about the current progress and upcoming challenges of the project.

## 2 GENERATION OF TRANSPLANTABLE PORCINE PANCREATIC ISLETS AND HUMAN BETA CELLS WITH REPORTER GENES FOR DIFFERENT IMAGING MODALITIES

Cell labelling with reporter genes such as fluorescent proteins (eGFP, dsRed, mCherry) allows tracking of transplanted cells fate.^14^, ^15^, ^16^ Unlike traditional indirect methods of cell labelling, such as superparamagnetic iron oxide (SPIO) and radionuclide labelling, reporter genes are inherited genetically (direct labelling) and can be used to monitor cell proliferation and survival for the lifetime of transplanted and their progeny cells. Current challenges and comparison of methods for stem cell tracking are reviewed.^17^, ^18^, ^19^, ^20^ In case of fluorescent reporters, changes in fluorescent signal indicate cell death or proliferation. These reporter genes cause the coloration of the cells expressing them and thus also allow the discrimination from histological sections as well as permit non‐invasive, real‐time tracking in vivo, but they are limited due to the penetration depths of visible light in the body.^21^ Near‐infrared fluorescent protein (iRFP) has been developed from the DrBphP bacterial phytochrome of *Deinococcus radiodurans* partially overcome this limitation, thus became a new prospect in the application of IFPs for protein labelling and in vivo tracking.^22^, ^23^

After careful consideration, for subsequent transplantation and imaging purposes we decided to develop the iRFP reporter driven by a pCAG promoter, both for porcine xenoislet and the hiPSC‐derived beta‐cell transplants. On the one hand, pCAG‐driven iRFP has already been reported to express reliably both in transgenic mice^24^ and in human cell lines implanted into mice.^25^ Tran et al (2014) measured carefully several physiological parameters of wild‐type control and pCAG‐iRFP transgenic mice and found no difference between them in body size, blood indices, reproductive performance or organ growth and morphology. Both studies reported a strong expression with well‐balanced intensity. On the other hand, the CAG promoter has been used successfully to drive the expression of several reporter genes in hiPSCs.^26^, ^27^, ^28^ These studies report a constitutive, stable and scalable reporter expression of the transgenes, both in pluripotent and differentiated cultures. Furthermore, the presence of the pCAG‐driven transgene did not alter the successful differentiation of the hiPSCs into other cell types. The same conclusions have been drawn both in case of transfection^26^ and single‐copy insertion into the AAVS1 safe harbour locus of hiPSCs.^27^, ^28^, ^29^ Luo et al compared the expression of eGFP under different regulation of promoters (CMW, EF1α, CAG), integrated into the AAVS1 locus of hiPS cell lines. pCAG‐driven expression yielded the best results: unlike the other two promoters, it showed no silencing and it allowed a stable, functionally strong expression, allowing detection both by microscopy and flow cytometry.^27^

**Figure 2:**
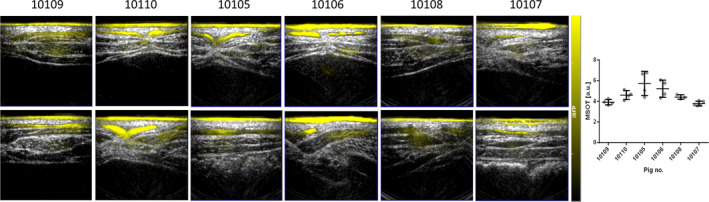
In vivo MSOT analysis of iRFP expression in CAG‐iRFP720 transgenic founder pigs. Three different regions were scanned at the belly region. Unmixed signals were pseudocoloured in yellow and overlayed to the corresponding ultrasound images. Notably, MSOT signal correlates very well to the FACS results of the iRFP fibroblasts showing strongest average signals for 10105 and 10106

**Figure 3:**
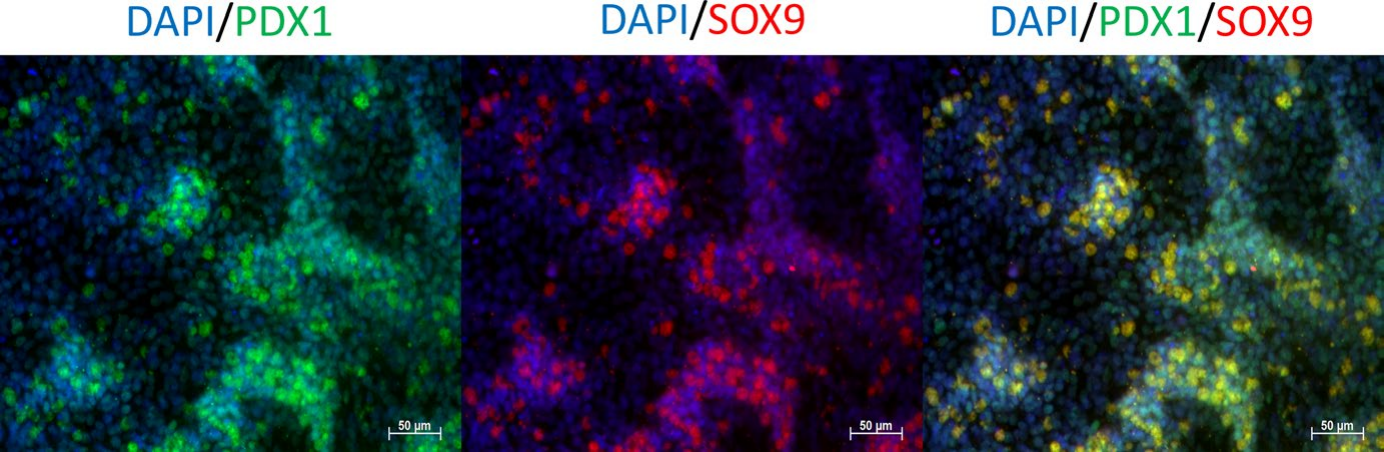
Representative immunofluorescent stainings of iPSC‐derived pancreatic progenitors with pancreatic progenitor markers PDX‐1, (in green) and, SOX9, (in red), nucleus were labelled with DAPI (in blue), (Scale bar, 50 μm)

Taken together, in our experiments pCAG has been chosen to drive the expression of the iRFP transgene. We have generated new transgenic pig lines expressing near‐infrared fluorescent protein, the iRFP720, which enables multispectral optoacoustic tomography (MSOT) to assess volumetric, quantitative differentiation of tissue and local evaluation of transplant viability (unpublished data). Culture conditions of porcine neonatal islet‐like cell clusters (NICCs) were optimized to guarantee high‐quality NICCs supply. In order to build up a breeding colony, pre‐existing genetically modified pig lines were introduced into a specified pathogen‐free pig facility —CiMM (Centre for Innovative Medical Models) by embryo transfer into specified pathogen‐free recipient sows, where embryos were generated by somatic cell nuclear transfer (SCNT) or in vitro fertilization.^30^ Thereby, provision of genetically optimized porcine pancreatic islets expressing LEA29Y^31^, ^32^ or INS‐eGFP expressing reporter islets^33^ suitable for human xenotransplantation processes is enabled. The iRFP720 expression of transgenic animals was verified by FACS analysis and subsequently by in vivo imaging using MSOT technology in transgenic pigs (Figure 2). Using CRISPR/Cas9 targeting technology in hiPSCs, we created iRFP reporter cell lines with the same construct as iRFP transgenic animals were generated. This cell line will enable the non‐invasive, optoacoustic detection of viable iPSC‐derived human beta cells^34^, ^35^ within a bioartificial pancreas, implanted into a pre‐clinical porcine model. Using directed differentiation methods, iPSCs were differentiated into pancreatic progenitor cells, which expressed the most relevant marker proteins of pancreatic progenitor cells (Figure 3), suitable for transplantations and further in vivo or in vitro maturation. Level of differentiation markers in iRFP expressing clone was similar to the maternal cell line assessed by RT‐qPCR analysis.

## 3 IN VIVO STUDIES IN PIGS—THE BIOARTIFICIAL PANCREAS (BAP)

Cell encapsulation currently represents the most promising method of avoiding autoimmune rejection of transplanted pluripotent stem cell–derived β cells, and therefore, it is an active area of worldwide research. This technique was introduced by Lim and Sun in 1980^38^ and has been continuously developed and adapted since then. Recent reviews summarized advances in the encapsulation methods.^36^, ^37^ This strategy has the potential to overcome graft rejection without the need for long‐term immunosuppressive medication, thus avoiding related side effects.^38^ Primordial for the success of such cellular transplantation therapy, the safety and functionality post‐implantation are key features of the bioartificial pancreas (BAP). Therefore, the BAP should be resistant enough to protect the encapsulated islets/cells from the host's immune system, but also to protect the receiving organism from the cells that could be of animal origin or stem cell–derived. Moreover, the BAP should allow optimal exchanges between the encapsulated cells and the receiving organism. Inadequate oxygen supply causes the gradual loss of cell mass and function, and this effect can be aggravated with encapsulation, thus pose one of the challenges in BAP development.^39^ To overcome this problem, several different experimental approaches have been tested such as the stimulation of vascularization growth prior to cell transplantation,^10^, ^40^, ^41^, ^42^, ^43^ the use of a hypoxia‐resistant cell line from Tilapia,^44^ microencapsulation,^45^ increased oxygen permeability of the encapsulating material.^46^ Incorporated refillable oxygen reservoir in the βAir device has already been tested in small and large animal models,^47^, ^48^, ^49^ and recently, in a clinical phase I study (Clinicaltrials.gov: NCT02064309) has been conducted to evaluate its safety.^34^ However, the βAir device requires daily refilling of the oxygen chamber thus patient needs to carry the equipment to refill the device which is inconvenient.^34^

**Figure 4:**
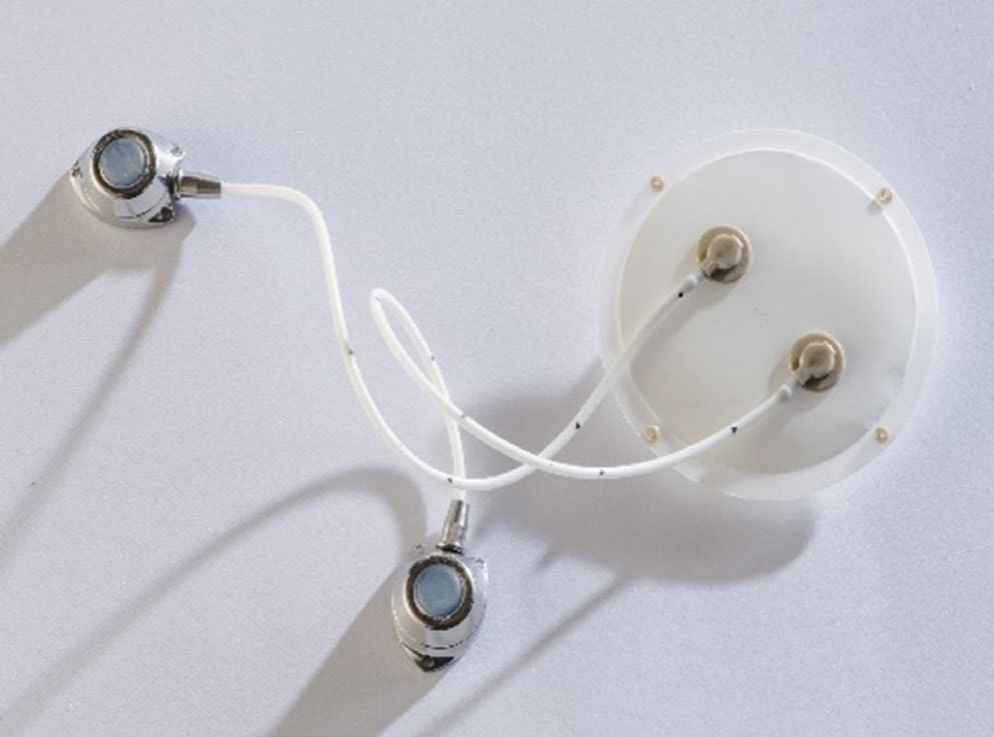
The MailPan^®^ (MAcroencapsulation of PANcreatic IsLets) device

The novel MailPan® (MAcroencapsulation of PANcreatic IsLets, see Figure 4) device^50^ uses of a permselective membrane allowing the passage of glucose and insulin, but impermeable to the immune system. Thanks to this property, no immunosuppressive treatment shall be needed. Innovative feature of MailPan^®^ is its “In” and “Out” implantable access ports that allow to fill or empty MailPan^®^, without the need of a surgery, whenever the cells might become non‐functional. Thanks to the in and out ports, the device can be implanted weeks before loading the cells which allow pre‐vascularizing the device prior the islet filling, which should improve their survival once filled in the MailPan^®^. This latest version of the MailPan^®^ is undergoing validation in late pre‐clinical phases. These steps together with the ongoing regulatory phases (Biocompatibility under ISO 10993, validation of sterility, etc) are primordial to obtain an authorization to enter clinical trials in human. The inlet and outlet channels where media and cells can be loaded and removed from the device without surgically removing it from the patient will allow repeated application of imaging agents during prolonged observations of several months, while keeping the benefits of encapsulation technology. This is a novel use of the device and would provide added value.

We implanted two MailPan^®^ devices for a 2‐month period, in one pig (Sus scrofa domesticus) to determine whether a subcutaneous implantation of MailPan^®^ device was feasible since MailPan^®^ devices were previously implanted in pre‐peritoneal site, which is not ideal for the MSOT imaging method, due to limitation of infrared laser light penetration into tissues, in the range of a few centimetres. Therefore, one MailPan^®^ device was implanted in subcutaneous site and the other one in pre‐peritoneal site to compare biointegration at both sites. The recipient pig was followed up during 2 months post‐implantation and was sacrificed. During the follow‐up, the implanted pig was in good conditions. Both recovered MailPan^®^ devices were intact at explantation after 2‐month follow‐up. In summary, the results showed the feasibility to implant MailPan^®^ device in subcutaneous as well as the integrity of the device. Depending on the maximum depth allowing accurate optoacoustic imaging, the MailPan^®^ device could be implanted subcutaneously which is a less deep site than pre‐peritoneal site.

## 4 DEVELOPMENT AND TESTING OF NANOMATERIALS FOR MULTIMODALITY ENHANCED IMAGING

In order to overcome the current sensitivity limitations of imaging technology, we take advantage of the unique characteristics of the nanoparticles, already established as innovative therapeutic and diagnostic agents.^51^, ^52^ Nanoparticles have the capacity to circulate in the body, as carriers loaded with small molecules such as drugs, diagnostic and imaging agents, minimizing the dispersion and degradation of such molecules and allowing them to reach more easily the target cells/organs in the body.^53^, ^54^, ^55^ The safe journey of the nanoparticles in the body requires prevention of their elimination by macrophages, which is possible by making them “stealth” using PEGylation or glycosylation at their surface.^56^ The dimension and shape^57^ of the nanoparticles are also very important for their kinetics and for overcoming biological barriers of the body. Another important issue is the capacity to accumulate at the target site (in our case on the beta cells), which requires a “targeting” strategy based on the concept that the nanoparticles must present at their surface a molecule able to recognize selectively the biological target, resulting in an adhesion on the surface or often favouring the internalization into the cells affected by a pathology.

Methods of intracellular labelling are disadvantageous as materials intracellularly might decrease cell viability. Combination of imaging technologies for higher sensitivity and resolution would require combination of methods such as nuclear medicine, magnetic resonance (MR) and MSOT, thus contrast agents allowing multimodality need to be produced and validated. Monitoring cell viability and function by distinguishing different types and states of cells is challenging task, as well. Many contrast agents produced with “traditional” methods work well; however, nanotechnological solutions can enhance the signal strength of single cells or small clusters of cells.^53^, ^58^

**Figure 5:**
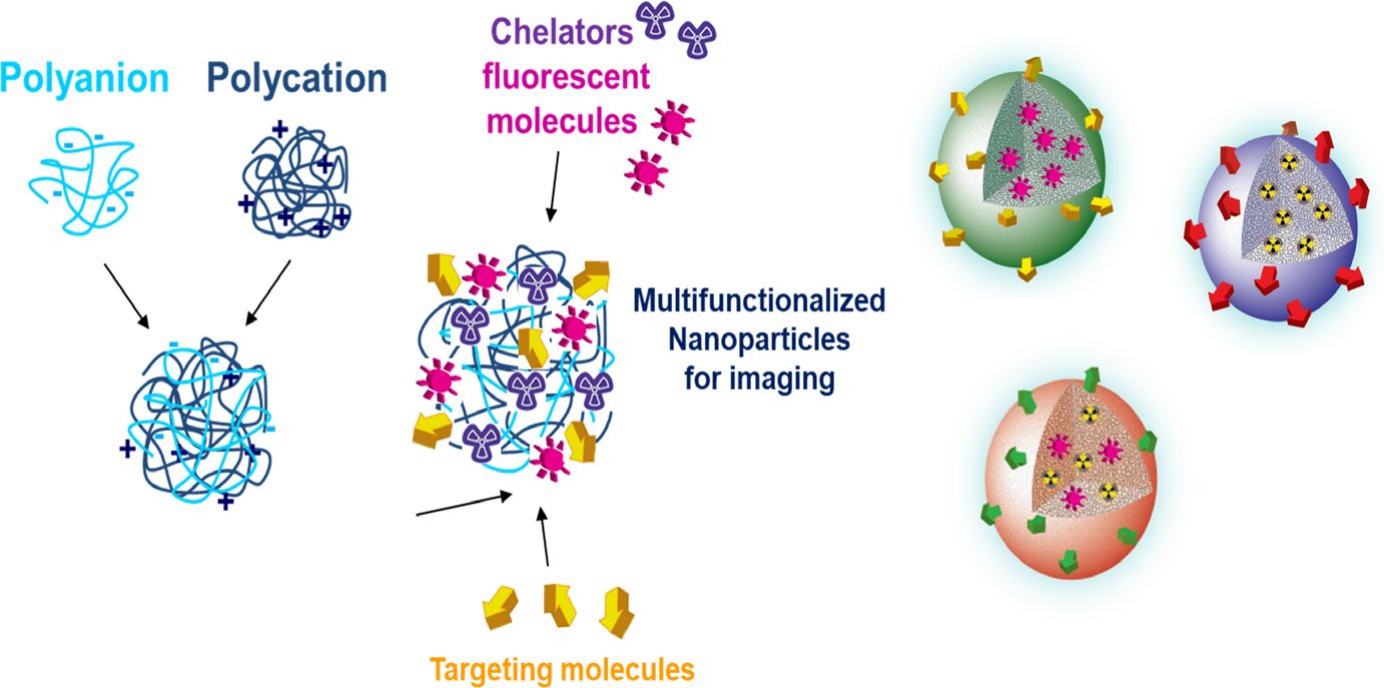
Strategy to generate multimodal nanoparticles. Polyanion and polycation nanoparticles are combined and multifunctionalized with specific biological recognition motifs (targeting molecules) to selectively label beta cells and diverse contrast agents for imaging purposes. Produced nanoparticles thereby hold targeting molecules on their surface while possess imaging molecules in the core as seen on the right side

Our aim was to create new agents for single‐photon emission computed tomography (SPECT), positron emission tomography (PET) and near‐infrared reflectance (NIR) labelling for optoacoustic imaging. We designed multifunctional nanoparticles (NPs) and engineered as specific biopolymer‐based nanotools in order to track the fate of transplanted cells and tissues (Figure 5). The highly flexible chemistry and structure of the nanotools allow conjugating of diverse contrast agents and create possibility of multimodal imaging and functionalizing of NPs with specific biological recognition motifs to selectively monitor beta‐cell survival and function in vivo. Our principal target of the NPs is the glucagon‐like peptide 1 receptor (GLP‐1R) which is expressed on the membranes of beta cells and has an important role in the regulation of insulin secretion.^59^, ^60^ The natural ligand, glucagon‐like peptide‐1 (GLP‐1) binds to the GLP1‐R with high affinity, but its half‐life in vivo is very short for the fine regulation of blood sugar level.^61^, ^62^, ^63^, ^64^ Current therapeutic approaches have utilized mimetics of GLP‐1, most notably exenatide, the synthetic form of the naturally occurring peptide exendin‐4 (Ex4).^65^

We have designed NPs for multimodality enhancing imaging, based on the combination of chitosan‐PGA, and detecting agents for MSOT, MRI and SPECT, respectively. The imaging NPs have been functionalized with Ex4 peptide, a ligand of GLP‐R thus we can selectively label beta‐cell population. The chitosan‐PGA NPs have been optimized by changing of the polymers molecular weight, proportion of compounds, crosslinking rates and NP’s size. The stability in different buffers (phys. salt, PBS) was tested. The polymeric components of NPs have been functionalized with IRDye® 800CW at BBS for MSOT detection; the NPs have been chemoselectively functionalized at the surface, through proper linkers, with Ex4 for targeting and with DOTA, a chelator of diagnostic metals (68‐Ga, Gd, 99‐Tc).

## 5 DEVELOPMENT OF NOVEL CLINICALLY APPLICABLE IMAGING APPROACHES

High‐resolution and sensitive whole‐body imaging in large animals and humans are areas where development is fast and promising, but the currently available imaging agents and clinical equipment are not sufficient to satisfy all of the needs of cell and tissue transplantation. Clinical imaging in humans allows larger size objects to be imaged, including head, limbs, arms or full body, on the expense of resolution and sensitivity. We are currently developing higher sensitivity equipment for pre‐clinical large animal models and humans, as well as developing automated or semi‐automated data analysis tools with new mathematical algorithms to enhance the information gained from the data acquired.

**Figure 6:**
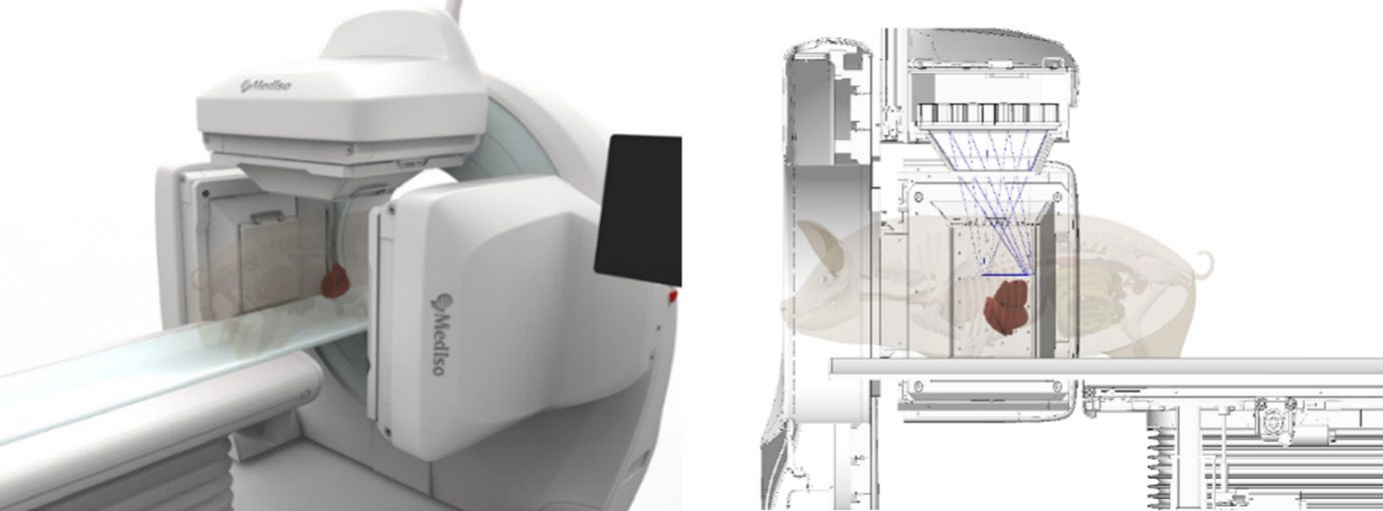
The AnyScan TRIO system with the new multi‐pinhole collimator design focusing on the pig's liver

The design of collimators has fundamental effect on the final performance of the SPECT imaging system. Multi‐pinhole collimators provide enough flexibility to effectively optimize the parameters of the camera for specific applications. We designed a new multi‐pinhole collimator, for imaging of transplanted islets inside certain regions of various sized pigs (*AnyScan TRIO system,* Figure 6). The central region of interest is the left liver lobe of the animals where most of the transplanted islets are expected to be located. Also, imaging the whole‐body radiopharmaceutical distribution is necessary. In contrast to standard clinical systems using parallel‐hole collimation, our novel system allows for quantitative analysis of image data and provides direct means for future translation to diagnostic imaging in humans. The collimator design process consists of multiple steps aiming for the translation of application needs to imaging apparatus. After summarizing the requirements for the new aperture like field‐of‐view, scan time and spatial resolution, a draft version is created, which goes through a multi‐level optimization from simple analytic formulas to complex Monte Carlo‐based simulations of the expected image quality. The final image quality of the optimized aperture is assessed by an exhaustive set of synthetic and anthropomorphic phantoms, and it is compared with the conventional imaging solutions.

## 6 IN VIVO IMAGING WITH NUCLEAR MEDICINE TECHNOLOGY PET AND SPECT IN COMBINATION WITH CT AND WITH MSOT

Finally, imaging of the transplanted pigs will be performed, applying our novel nanotools, reporter pig and cell lines. In order to establish PET/CT and SPECT/CT imaging of pigs, there are several prerequisites, which need to be fulfilled. These include approvals in the context of animal welfare and radiation safety. Furthermore, the installation of our new SPECT/CT device is currently being developed near to the pig facility.

In order to create the imaging protocols for pigs, we prepared PET/CT measurements on pigs using fluorodeoxyglucose. Using this well‐established tracer for measuring glucose metabolism before labelled, exendin derivatives would become available in iNanoBIT, will yield quantitative measurements of glucose metabolism. Importantly, basic information on animal welfare during PET/CT scanning can be gained as a prerequisite for optimization of the planned exendin scans and subsequent detailed description for the application to achieve approval by the authorities. This study is also essential to establish logistics and radiation safety procedures in animal transport, housing, anaesthesia, as well as monitoring during PET/CT scanning and wake‐up phase after the imaging session. In addition, these scans provide specific information on PET sensitivity and organ sizes in the pig.

## 7 CONCLUSIONS

The potential socio‐economic impact of the regenerative medicine solutions for T1D is high. According to the IDF Diabetes Atlas globally, 382 million people had diabetes in 2013, half of it undiagnosed. Type 2 diabetes makes up about 90% of the cases. A diabetes epidemic is underway: estimates have put the numbers as high as 550‐600 million in 2013. According to the World Health Organization (WHO) direct, healthcare costs of diabetes‐related illnesses range from 2.5% to 15% of a country annual healthcare budget, depending on local diabetes prevalence and the sophistication of the treatment available, with a predicted increase from 7%‐13% up to 40% in high prevalence countries (WHO: from IDF Diabetes Atlas, 2003). Globally, 12% of the health expenditures and USD 1330 per person are anticipated to be spent on diabetes in 2020. There are indirect costs associated with diabetes: low productivity due to the inability to work, sickness, absence, disability, premature retirement or premature death. These social costs and impact may very well be even greater than all the other costs (30%). Projecting these to the present and to global level, we estimate that the global cost of T1D in 2013 was USD 50Bn, including global healthcare spending (medical costs) on T1D of USD 35 Bn and indirect cost of USD 15 Bn. This is the value an efficient regenerative treatment could potentially save to healthcare authorities worldwide.

The iNanoBIT project is aimed to provide the currently missing toolbox for pre‐clinical/clinical testing for safe transplantation of regenerative medicinal cellular and tissue products, currently in pre‐clinical and clinical trials. The key technical components under development, such as porcine islet cells/human beta cells, encapsulation device and safety arrays, have the capacity to provide new and innovative solutions to major medical and societal problems such as (a) the lack of cell supply for human transplantation in T1D, (b) the need for chronic immunosuppression following islet transplantation and (c) complicated surgical procedures. Indeed, this innovative therapy could open the way to a better treatment of T1D patients who are not even considered for human islet transplantation.

The source of porcine cells or hiPSCs would be, effectively, infinite and limited only by the animal welfare guidelines, safety and QC procedures. Since no systemic immunosuppression would be needed (in case of closed BAP encapsulated islets/beta cells), the recipient immune system being intact, would significantly decrease the potential risk of infections, and zoonosis when using xenogeneic cells, or that of tumours from stem cells or patient own cellular mutations and moreover would give access to treatments for a more fragile younger and older patient population. Major efforts are needed towards a scaling‐up of the production of transplantable cells/islets on a cost‐effective manner and generating data towards regulatory acceptance of the novel ATMPs.

## CONFLICT OF INTEREST

None.

## AUTHOR CONTRIBUTION

AD, JK, EW, PB, C‐TB CC, FN, GN, PG, EP and NB conceived and planned the experiments. AS, SM, JK, EW, PB, CC and EK contributed generating iRFP expressing transgenic pigs or differentiated beta cells from hiPSCs. EP, LR and FN contributed to design and produce nanoparticles for different imaging modalities. NB, GN, YQ, SZ and AW contributed in imaging equipment development. AD took the lead in writing the manuscript. All authors read and approved the final version of the paper.

## Data Availability

The authors confirm that the data supporting the findings of this study are available within the article.
